# Consult the Doctor

**Published:** 1921-04-23

**Authors:** 


### CONSULT THE DOCTOR.

Several members of the medical profession have expressed great interest in an article which recently appeared in the Public Well-Being Section of The Hospital, in which we pointed out the close connection probably existing between the modern Labour strike and the worker's liability to sickness, and suggested that this was essentially a matter requiring medical co-operation. In reply to a request to amplify this suggestion, we have sufficient space only to indicate the lines on which the Government or any other arbiters in a dispute about wages might gain knowledge by seeking the testimony of doctors.

In the first place nearly every industrial disagreement concerns wages, and in almost every case nowadays the question is debated on the basis of the standard of living. What is an adequate standard of living? No common ground has been reached on this point by any of the disputants in any of these wage problems, and the one profession in the country which could offer precise, scientific, practical evidence is never invited to testify. There is no reason why an authoritative medical opinion should not be asked from a representative body on the general question; and in the case of separate disputes, relating to a particular trade in a particular district, the advice of an impartial medical referee specially appointed because of his expert knowledge of local conditions, either in the industry or the district, would save endless discussion and suffering, and might not only assist the claims of Labour justly framed, but supply employers with information that would be invaluable to them in the more economic organisation of their industry.

Moreover, it should be the business of the Government to obtain from the medical men a carefully considered reckoning of the effect of an inadequate wage on the health and productive efficiency of the worker and his family; and all parties perhaps would be much more eager to avoid the palpable misfortune of the strike policy if they could be made aware of the actual loss to the community represented by diminished vitality and lowered working efficiency in the human machine.

